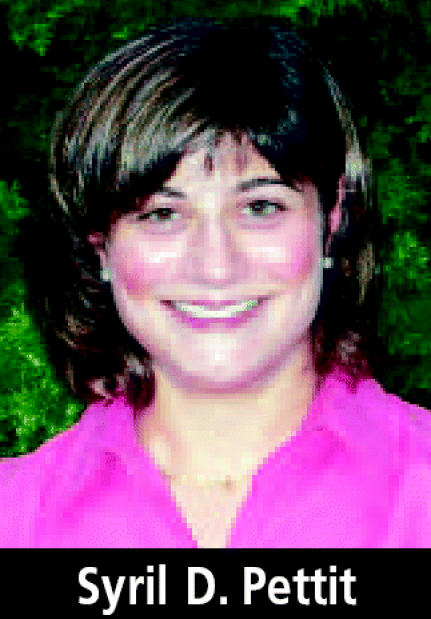# Guest Editorial: Toxicogenomics in Risk Assessment: Communicating the Challenges

**DOI:** 10.1289/ehp.112-1277120

**Published:** 2004-08

**Authors:** Syril D. Pettit

**Affiliations:** Health and Environmental Sciences Institute, International Life Sciences Institute, Washington, DC, E-mail: spettit@ilsi.org

In 1999 the membership of the International Life Sciences Institute (ILSI) Health and Environmental Sciences Institute (HESI) formed a multisector consortium to address challenges associated with the integration of genomics data into risk assessment (Pennie et al. 2004). Following its formation, the HESI Committee on the Application of Genomics to Mechanism-Based Risk Assessment identified several key hurdles. These included a lack of publicly available toxicogenomics databases, a lack of validation of available technologies, questions concerning the comparability of different technical platforms and how transcription products relate to toxicity, and uncertain regulatory applications.

In 2004 we have seen considerable progress in many of the areas mentioned above, particularly in our technical ability to execute microarrays and to analyze and interpret the resultant data. The experimental program of the HESI Genomics Committee clearly demonstrated that it is possible to replicate data on biologic pathways across laboratories and technical platforms (Kramer et al. 2004; Ulrich et al. 2004). The committee’s work also revealed the need to interpret modulations in gene expression on microarrays in the context of a broader biologic data set (e.g., clinical chemistry, histopathology). Additionally, within the United States, the recent release of draft regulatory guidance from the U.S. Food and Drug Administration (FDA 2003) on the use of pharmacogenomics data in risk assessment and the release of a white paper from the U.S. Environmental Protection Agency (U.S. EPA 2004) on potential regulatory applications of genomics data have further focused potential applications. However, the routine application of genomics to preclinical risk assessment has not yet been accepted universally. Why?

The efforts of the HESI Committee on Genomics regarding experimental collaboration and toxicogenomics database development (Mattes et al. 2004) suggest that some of the greatest outstanding challenges relate to effective communication across key user groups. It is critical that regulated industries share with the regulatory community the focus of their current approaches to the use of genomics. For example, are microarray data used primarily for early screening or for researching mechanisms of toxicity and and under what circumstances? Additionally, open information exchange regarding typical means of data analysis and presentation is needed. This exchange, which has been initiated via several multisector forums including HESI, will help ensure that a common understanding of the technology’s practical strengths and limitations is reached and allow genomics to be applied more effectively to safety assessment. Discussions concerning the interpretation of patterns of change in gene expression in relation to other biologic end points will provide critical context for determining the suitability of a data set for risk evaluation. Consensus as to when changes in gene expression via microarray represent definitive biomarkers of effect is also needed. Until these conditions are clarified, the utility of genomics for classifying effects of concern will remain debatable.

The risk assessment community is also striving both to harness the collective power of publicly available data sets and to facilitate exchange of single data sets for safety evaluation. As such, numerous formats for the capture and exchange of microarray and toxicology data have become available and/or are under development (Mattes et al. 2004). Diversity of approach is not in itself problematic and clearly has its benefits. However, the development of flexible and comprehensible data exchange platforms that meet the needs of multiple user groups is essential for routine exchange of toxicogenomics data.

The HESI Committee on Genomics looks forward to an ongoing role as a multi-stakeholder consortium committed to facilitating discussion on the scientifically sound use of genomics for risk assessment.

## Figures and Tables

**Figure:**